# High pretreatment level of soluble interleukin-2 receptor is a robust prognostic factor in patients with follicular lymphoma treated with R-CHOP-like therapy

**DOI:** 10.1038/bcj.2017.96

**Published:** 2017-09-29

**Authors:** Y Kusano, M Yokoyama, Y Terui, N Inoue, A Takahashi, H Yamauchi, N Tsuyama, N Nishimura, Y Mishima, K Takeuchi, K Hatake

**Affiliations:** 1 Department of Hematology Oncology, Cancer Institute Hospital, Japanese Foundation for Cancer Research, Tokyo, Japan; 2 Division of Pathology, The Cancer Institute, Japanese Foundation for Cancer Research, Tokyo, Japan; 3 Pathological Project for Molecular Target, The Cancer Institute, Japanese Foundation for Cancer Research, Tokyo, Japan

Follicular lymphoma (FL) is one of the most common subtypes of non-Hodgkin lymphoma. Indolent lymphomas including FL are generally considered as incurable, which impacts the treatment goals including progression-free survival (PFS) and time to next treatment (TTNT). Rituximab is a human-mouse chimeric immunoglobulin G monoclonal antibody and show a high impact on PFS and TTNT in FL. In particular, rituximab maintenance (RM) is highly effective.^1, 2^ FLIPI and FLIPI2 that are useful prognostic models were devised before the rituximab era and invented by a retrospective study, respectively. Thus, independent prognostic biomarkers of FL in rituximab era remain unknown.

Interleukin-2 receptor (IL-2R) expressed on the cell membrane of lymphocytes has three known glycoprotein chains, that is, α, β and γ, which are known to bind to the ligand independently and have a role in activation and proliferation of lymphocytes.^3^ Soluble IL-2R (sIL-2R), an IL-2Rα isoform released from lymphoma cells is known to be associated with poor prognosis of non-Hodgkin lymphoma including DLBCL^4, 5^ and with tumor-related immunosuppression. Absolute CD4^+^ T-cell count is a robust prognostic marker in B-cell lymphomas,^6, 7^ and regulatory T cells (Treg), a specific type of CD4^+^ T cells, inhibit the production of cytokines released from CD4^+^ T cells and the proliferation of CD4^+^ T cells themselves.^8^ IL-2, which is released from activated T cells, is essential for Treg to develop and function.^9^ sIL-2R promotes T-cell differentiation toward inhibitory Treg, *in vitro*, rather than Th1 and Th17 cells in FL.^10^ Thus, we investigated whether the prognostic power of sIL-2R was comparable to those of other previously identified prognostic factors in FL.

The medial records of all untreated FL patients who were diagnosed according to the 2008 WHO classification at the Cancer Institute Hospital, Tokyo, Japan between 2005 and 2014 were retrospectively accessed. Patients with grade 1, 2 or 3A FL, who achieved complete remission or partial response, were included in this study. Exclusion criteria were grade 3B FL and histological transformation. All patients were staged according to the revised International Working Group criteria.^11^ The institutional review board of the hospital approved the study, which was conducted in accordance with the Declaration of Helsinki.

All patients received rituximab, cyclophosphamide, vincristine, prednisolone with or without doxorubicin (R-CVP/R-CHOP).^12, 13^ Patients who achieved complete remission/partial response were candidates for RM. Patients, who signed informed consent form were provided RM,^2^ whereas those patients who declined RM were followed until disease progression.

Primary endpoint was PFS, and secondary endpoints were TTNT and overall survival (OS).^11^ The cut-off value for sIL-2R to predict a relapse was determined by the receiver operating characteristic curve. Survival endpoints were evaluated using the Kaplan–Meier method and Cox proportional hazards model. Differences among the results of comparative tests were considered significant if two-sided *P* values were <0.05. All statistical analyses were performed using EZR.

A total of 219 patients with FL who achieved complete remission/partial response after R-CVP/R-CHOP were included in the current study (Supplementary 1). Baseline characteristics were as follows: age >60 of 105 (47%), stage III/IV of 174 (79%), ⩾four nodal lesions of 118 (54%), grade 3A of 33 (15%), bone marrow involvement (BMI) of 84 (40%), and bulky tumor (>6 cm) of 43 (20%), elevated lactate dehydrogenase (LDH, >245 U/l) of 36 (16%), decreased hemoglobin (<12 g/dl) of 27 (12%), increased β2 microglobulin (β2MG, >2 mg/dl) of 83 (37%), high FLIPI of 66 (30%) and high FLIPI2 of 50 (23%) patients. R-CVP and R-CHOP were provided to 151 (69%) and 68 (31%) patients, respectively. RM was administered to 169 patients (77%) for a median duration of 1.6 years. At a median follow-up time of 74.2 months, 58 (26%) patients suffered a relapse of FL and 55 (25%) patients received salvage chemotherapy. As salvage chemotherapies, bendamustine plus rituximab (BR) was the most implemented regimen (24; 44%). BR showed particularly high efficacy including overall response rate of 96% and complete remission rate of 83%. In agreement with the results of previous reports, RM prolonged PFS and TTNT significantly but did not upgrade OS (Supplementary 2).

Median pretreatment sIL-2R level was 871 U/ml (range, 115–13700). Median sIL-2R level at diagnosis in the non-relapse group (*n*=120) was significantly lower than that in the relapse group (Supplementary 3A, *n*=99; 1414 and 2605 U/ml, respectively; *P* <0.001). The receiver operating characteristic curve determined 1070 U/ml as a satisfactory cut-off value to predict a relapse (Supplementary 3B, area under the curve, 0.7; specificity, 0.67; sensitivity, 0.77; 95% CI, 0.62–0.78). Using this cut-off value, patients were classified into 120 (55%) patients with low sIL-2R and 99 (45%) patients with high sIL-2R. Stage III/IV, ⩾four nodal lesions, BMI, bulky tumor, elevated LDH, increased β2MG, high FLIPI and high FLIPI2 were significantly associated with high sIL-2R levels. Six-year PFS (51.1% (95% CI, 39.8–61.3) versus 84.0% (95% CI, 74.4–90.2), Figure 1a, *P*<0.0001) and TTNT (49.4% (95% CI, 37.4–60.3) versus 81.1% (95% CI, 69.2–88.8), Figure 1b, *P*<0.0001) were significantly lower in the high sIL-2R group. Six-year OS was not different statistically (96.2% (95% CI, 90.2–98.6) and 89.9% (95% CI, 80.7–94.9), Figure 1c, *P*=0.06). When both sIL-2R (high or low) and RM (yes or no) were considered as covariates, 6-year PFS and TTNT of the non-RM/high sIL-2R group were significant lower than those of other three groups (Figure 1d and e). The addition of high sIL-2R as a covariate led to a reduction in PFS and TTNT not only in the non-RM group (*P*<0.001) but also in the RM group (*P*<0.001). Six-year PFS and TTNT of the RM/high sIL-2R group were almost the same as those of the non-RM/low sIL-2R group. No influence was observed in OS (Figure 1f).

Univariate analyses were conducted using the following variables to determine independent prognostic markers for FL in this cohort (Table 1): variables of FLIPI, high FLIPI, variables of FLIPI2, high FLIPI2, male gender, no RM and high sIL2R at diagnosis. Multivariate analysis 1 using the variables selected by univariate analysis except high FLIPI and high FLIPI2 revealed that high sIL-2R was an independent prognostic marker in this cohort. Both multivariate analysis 2, which included high FLIPI, high sIL-2R, and no RM and multivariate analysis 3, which included high FLIPI2, high sIL-2R and no RM, demonstrated that high sIL-2R was an independent prognostic factor. No RM was also determined as significant, whereas neither high FLIPI nor high FLIPI2 had significant impact on PFS in this cohort.

In this cohort, we identified that high sIL-2R level before treatment was associated with worse PFS in FL patients treated with R-CVP/R-CHOP. High sIL-2R level affected only PFS and TTNT, which was because BR was provided to most, if not all, patients, who suffered a relapse. Recently, BR was reported for its efficacy in two non-inferiority studies,^14, 15^ suggesting that BR salvaged and equalized OS of the high sIL-2R group. A cut-off value for sIL-2R of 1070 U/ml in the present study was twice the normal upper limit of sIL-2R. Two previous papers used the sIL-2R cut-off value of 1000 U/ml to predict prognosis in DLBCL,^4, 5^ suggesting that a sIL-2R level >1000 U/ml at diagnosis is crucial for not only DLBCL but also FL for unknown reasons.

Multivariate analysis in this cohort showed no statistical association of variables of either FLIPI or FLIPI2 and high-risk score of these prognostic models with PFS, suggesting that not merely high level of sIL-2R but not receiving RM can robustly predict poor PFS. Despite both FLIPI and FLIPI2 being skillful at predicting PFS, these models did not weigh the efficacy of RM. The results of multivariate analyses in the present study suggest that another new prognostic model is required for FL population where majority of them were provided RM.

Not receiving RM showed significant negative influence on PFS, in particular the high sIL-2R level group in this cohort. Patients with high-tumor burden are known as good candidates for RM.^1^ Population with high sIL-2R in this cohort resembles that with high-tumor burden,^1^ which was compatible because high sIL-2R reflects high activity of lymphoma. Therefore, high sIL-2R level at diagnosis may be utilized as a surrogate marker to identify FL patients treated with R-CVP/R-CHOP who should be recommended RM.

One limitation of the present study is that it is a retrospective study that included a relatively small number of patients. Thus, further large prospective studies are necessary to validate the results and the implications of the current study, which showed that high sIL-2R was associated with a poor prognosis of FL treated with R-CVP/R-CHOP and that FL with high sIL-2R level was the population to recommend RM strongly.

In conclusion, we identified that high sIL-2R level at diagnosis was an independent prognostic marker for FL treated with R-CVP/R-CHOP in this cohort. Furthermore, a high pretreatment sIL-2R level was associated with a poor PFS in FL patients, in particular, if they had no RM.

## Figures and Tables

**Figure 1:**
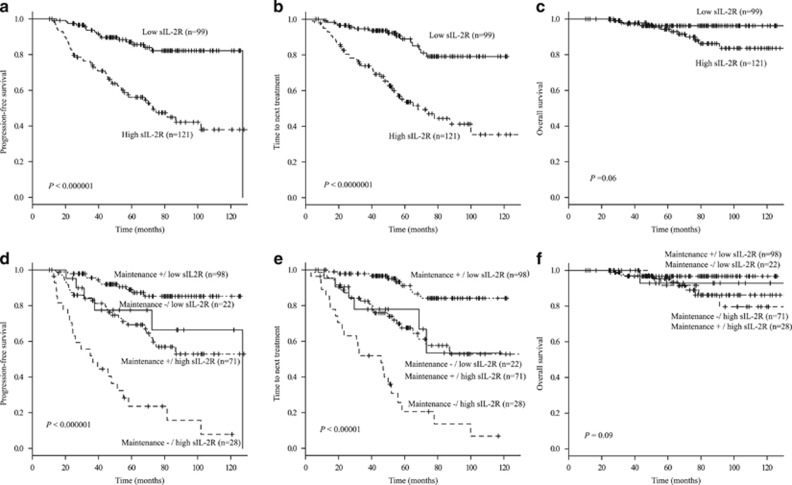
Kaplan–Meier curves showing progression-free survival, time to next treatment and overall survival in groups stratified according to the soluble interleukin-2 receptor level and RM therapy. At the median follow-up of 74.2 months, 6-year progression-free survival (PFS (**a**)) and time to next treatment (TTNT (**b**)) rates of the high sIL-2R group were significantly worse than that of those of the low sIL-2R group. There was no difference in overall survival (OS (**c**)). If both sIL-2R (high or low) and RM (yes or no) were considered as covariates, six-year PFS were 84.8%, 78.4%, 58.5% and 48.1% in the RM/low sIL-2R (*n*=98), non-RM/low sIL-2R (*n*=22), RM/high sIL-2R (*n*=71) and non-RM/high sIL-2R (*n*=28) groups, respectively ((**d**) *P*<0.0001). Six-year TTNT were 83.7%, 69.3%, 50.8% and 49.1% in the RM/low sIL-2R, non-RM/low sIL-2R, RM/high sIL-2R and no maintenance/high sIL-2R groups, respectively ((**e**) *P*<0.0001). There was no difference in OS ((**f**) *P*=0.09).

**Table 1:** Univariate and multivariate analyses to determine factors predicting progression-free survival in patients with follicular lymphoma

*Variables*	*Univariate*		*Multivariate 1*		*Multivariate 2*		*Multivariate 3*	
	*HR, 95% CI*	P *value*	*HR, 95% CI*	P *value*	*HR, 95% CI*	P *value*	*HR, 95% CI*	P *value*
Age >60 years	1.0, 0.6–1.6	0.96	—	—	—	—	—	—
Male sex	1.2, 0.8–2.0	0.41	—	—	—	—	—	—
LDH >245 U/l	2.5, 1.4–4.3	< 0.01	1.1, 0.6−2.1	0.68	—	—	—	—
Stage III/IV	2.9, 1.3–6.4	< 0.01	1.8, 0.7−4.8	0.25	—	—	—	—
Involvement nodal sites ⩾5	1.8, 1.1–3.1	0.02	1.2, 0.7−2.2	0.45	—	—	—	—
Hemoglobulin <12 g/dl	1.3, 0.7−2.6	0.44	—	—	—	—	—	—
No rituximab maintenance	3.6, 2.1−5.8	<0.000001	3.7, 2.2−6.2	<0.00001	3.3, 2.0−5.5	<0.00001	3.3, 2.0−5.4	<0.00001
Bone marrow involvement	1.4, 0.9−2.3	0.69	—	—	—	—	—	—
sIL-2R >1070 U/ml	4.7, 2.6−8.5	<0.000001	2.5, 1.2−5.0	0.01	3.4, 1.9−6.1	<0.0001	3.3, 1.9−6.3	<0.0001
β2MG >2 mg/dl	3.3, 2.0−5.5	<0.00001	1.8, 1.0−3.3	0.05	—	—	—	—
Tumor diameter >6 cm	2.4, 1.4−4.1	<0.01	1.5, 0.8−2.7	0.17	—	—	—	—
High FLIPI (score ⩾3)	2.0, 1.2−3.3	<0.01	—	—	1.4, 0.8−2.3	0.26	—	—
High FLIPI2 (score ⩾3)	2.1, 1.3−3.6	<0.01	—	—	—	—	1.2, 0.7−2.1	0.48

Abbreviations: β2MG, beta-2 microglobulin; CI, confidential interval; FLIPI, follicular lymphoma international prognostic index; HR, hazard ratio; IgG, immunoglobulin G; LDH, lactate dehydrogenase; sIL-2R, soluble interleukin-2 receptor.
