# Supplementary material for: High pretreatment level of soluble interleukin-2 receptor is a robust prognostic factor in patients with follicular lymphoma treated with R-CHOP-like therapy
Source: Blood Cancer J. 2017 Sep 29;7(9):e614–. doi: 10.1038/bcj.2017.96 (PMC5709758; doi:10.1038/bcj.2017.96)
Supplement: Supplementary Figures and Table Legends [file bcj201796x4.docx]

**Legends of supplementary figures and table**

Supplementary table 1.

Baseline characteristics of patients with low and high soluble interleukin-2 receptor levels.

The cut-off value for sIL-2R was 1070 U/ml.

Abbreviations: β2MG, beta 2 microglobulin; FLIPI, follicular lymphoma international prognostic index; LDH, lactate dehydrogenase; sILR-2, soluble interleukin-2 receptor

Supplementary figure 1

Kaplan-Meier curves showing survival rates in the maintenance and no maintenance groups.

Kaplan-Meier curves showing event-free survival rates in the rituximab maintenance (n =169) and no maintenance (n = 50) groups. At a median follow-up of 6 years, the progression-free survival (PFS, A) and time to next treatment (TTNT, B) rates in the rituximab maintenance group were significantly better than those in the no maintenance group (PFS, 75.1% versus 44.1%, *P* < 0.0001; TTNT, 75.4% versus 40.1%, *P* < 0.0001). Overall survival (C) was not significantly different between the rituximab maintenance and no maintenance groups (93.1% versus 91.9%, *P* = 0.35).

Supplementary figure 2.

Relationship between pretreatment soluble interleukin-2 receptor level and relapse events in the study cohort.

A. Pretreatment soluble interleukin-2 receptor (sILR-2) levels in the non-relapse and relapse groups. Pretreatment sIL-2R levels were significantly higher in the relapse group. The mean sIL-2R levels at diagnosis were 1414 U/ml and 2605 U/ml in the non-relapse (n = 120) and relapse (n = 99) groups, respectively (*P* < 0.001).

B. Receiver operating characteristic (ROC) curve determined that the cut-off value for sILR-2 level at diagnosis as 1070 U/ml to predict FL relapse in the study cohort (area under the ROC curve, 0.7; specificity 0.67; sensitivity 0.77; 95% confidence interval, 0.62–0.78).
